# Thermoelectric Inversion in a Resonant Quantum Dot-Cavity System in the Steady-State Regime

**DOI:** 10.3390/nano9050741

**Published:** 2019-05-14

**Authors:** Nzar Rauf Abdullah, Chi-Shung Tang, Andrei Manolescu, Vidar Gudmundsson

**Affiliations:** 1 Physics Department, College of Science, University of Sulaimani, Sulaimani 46001, Kurdistan Region, Iraq; 2 Komar Research Center, Komar University of Science and Technology, Sulaimani 46001, Kurdistan Region, Iraq; 3 Department of Mechanical Engineering, National United University, 2, Lienda, Miaoli 36063, Taiwan; cstang@nuu.edu.tw; 4 School of Science and Engineering, Reykjavik University, Menntavegur 1, IS-101 Reykjavik, Iceland; manoles@ru.is; 5 Science Institute, University of Iceland, Dunhaga 3, IS-107 Reykjavik, Iceland; vidar@hi.is

**Keywords:** thermoelectric transport, quantum dot, QED, quantum master equation, electro-optical effects

## Abstract

We theoretically investigate thermoelectric effects in a quantum dot system under the influence of a linearly polarized photon field confined to a 3D cavity. A temperature gradient is applied to the system via two electron reservoirs that are connected to each end of the quantum dot system. The thermoelectric current in the steady state is explored using a quantum master equation. In the presence of the quantized photons, extra channels, the photon replica states, are formed generating a photon-induced thermoelectric current. We observe that the photon replica states contribute to the transport irrespective of the direction of the thermal gradient. In the off-resonance regime, when the energy difference between the lowest states of the quantum dot system is smaller than the photon energy, the thermoelectric current is almost blocked and a plateau is seen in the thermoelectric current for strong electron–photon coupling strength. In the resonant regime, an inversion of thermoelectric current emerges due to the Rabi-splitting. Therefore, the photon field can change both the magnitude and the sign of the thermoelectric current induced by the temperature gradient in the absence of a voltage bias between the leads.

## 1. Introduction

Thermoelectric transport through nanoscale systems has been studied experimentally [1,2] and theoretically [3,4,5], with the aim of controlling heat flow and harvesting thermal energy. Special interest has been placed on the characteristics of thermoelectrics of a quantum dot (QD) in the Coulomb blockade (CB) regime, both in weakly and strongly coupled QD devices [6,7,8]. Several approaches have been used to increase the efficiency of such devices. The efficiency of the QD system has been studied considering either the Coulomb interaction in two- or multi-level QD using non-equilibrium Green’s function (NEGF) methods [9] or the electron–phonon contribution [10]. A general formalism modeling the heat current in a lead–QD–lead system by NEGF has been proposed, and it has been shown that the heat current could be very high in the Coulomb blockade regime in which the thermoelectric current is very low due to the Coulomb blockade effect [11].

Besides the conventional thermoelectric structures, spintronic devices have been employed to enhance and control the efficiency of the thermoelectric transport using the spin degree of freedom in addition to charges [12,13]. To build a spintronic nanoscale system, spin polarized electrons have to be considered. One approach has been to take into account the Rashba-spin orbit coupling in the dot system [14,15] or assume ferromagnetic lead-based spintronic devices. In both cases, the spin effects can cause an increase in the figure of merit and in thermal conductance, which are significant in controlling the performance of nanodevices.

Another technique to control thermoelectric efficiency is to use a photon field. The thermoelectric current between two nanodevices, mediated by quantized photon field, can be controlled with an intermediate quantum circuit leading to the building of a mesoscopic photon heat transistor [16]. The proposed thermal quantum transistor could be utilized to develop devices such as a thermal modulator and a thermal amplifier in nanoscale systems [17]. Furthermore, it has been shown that heat can be transferred by electromagnetic radiation at a very low temperature when the phonons are frozen out [18], and the photon field can significantly modify the magnitude and the sign of the electrical bias voltage induced by the temperature gradient [19], which has important roles in the thermal amplifier.

Based on the aforementioned investigations, we study thermoelectric transport through a QD system coupled to a photon cavity, where the QD system is either in resonance or off-resonance with the photon field. A Markovian version of a Nakajima–Zwanzig generalized master equation is used to study the transport characteristics of the total system [20,21,22]. In previous publications, we have reported that the transient thermoelectric [23] and heat [24,25] currents can be modulated using a cavity photon field with even a single photon [26]. The influences of the photon polarization and the electron-photon coupling strength on the transient thermospin current and spin-dependent heat current in different systems have been demonstrated [15,27,28].

In this work, we theoretically investigate the thermoelectric current through a QD system in the steady-state regime. The effects of a photon field, such as polarization and electron-photon coupling strength, on the thermoelectric current are shown. A thermoelectric current oscillation “peak” is observed due to the photon-assisted tunneling processes. In addition, a thermoelectric current plateau in the off-resonance regime and current inversion in the resonant regime are found.

## 2. Modeling and Formalism

**Figure 1:**
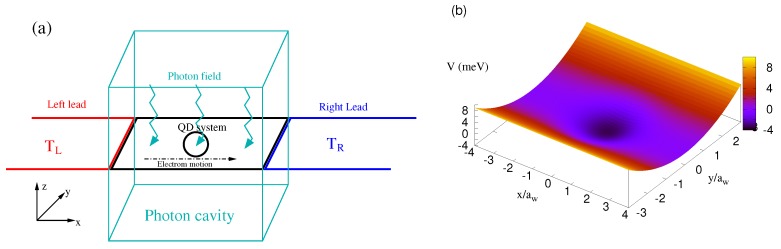
(**a**) Schematic diagram showing the quantum dot (QD) system (black) connected to the leads or the electron reservoirs, where the temperature of the left electron reservoir ($T_{L}$) (red) is higher than the temperature of the right electron reservoir ($T_{R}$) (blue). The cyan zigzags represent the photon field in the cavity (cyan rectangle). (**b**) The potential of the QD system to be connected diametrically to the left and right leads in the *x*-direction.

In this section, the model and the theoretical formalism used to calculate the thermal properties of the system are presented. We assume a quantum dot embedded in a two dimensional quantum wire, and the QD system is coupled to two semi-infinite leads from both ends, as is shown in Figure 1a. The cyan zigzag arrows indicate the photon field inside the photon cavity (cyan rectangle) coupled to the QD system. The temperature of the left lead ($T_{L}$) (red color) is considered to be higher than that of the right lead ($T_{R}$) (blue color). The contact regions couple the QD system and the leads. Figure 1b shows the potential of the quantum dot embedded in the two-dimensional quantum wire in the xy-plane. The electrons are driven through the QD system in the *x*-direction by the thermal bias.

The potential used to define the QD system can be represented as

(1) $$\begin{array}{c} V_{\mathrm{QD}}\left(x,y\right)=\left[\frac{1}{2}m^{*}\Omega_{0}^{2}y^{2}+eV_{p}+V_{0}\exp\left(-\beta_{x}^{2}x^{2}-\beta_{y}^{2}y^{2}\right)\right]\times \theta \left(\frac{L_{x}}{2}-\left|x\right|\right), \end{array}$$

where $\Omega_{0}$ is the electron confinement frequency due to the lateral parabolic potential, $m^{*}$ refers to the effective mass of the electrons, $V_{p}$ indicates the plunger-gate voltage that shifts the energy states of the QD system with respect to the chemical potential of the leads, and θ is the Heaviside unit step function with the length of the quantum wire $L_{x}=150$ nm. Here, we assume $V_{0}=-3.3$ meV and $\beta_{x}=\beta_{y}=0.03$ nm${}^{-1}$, determining the diameter of the QD.

The QD system is hard-wall confined in the *x*-direction and parabolically confined in the *y*-direction. In two-dimensional systems, the Hamiltonian can be described by [29,30,31]

(2) $$\begin{array}{cc} {\hat{H}}_{S} & =\int d^{2}r\;{\hat{\psi }}^{†}\left(r\right)\left[\frac{1}{2m^{*}}{\left(\frac{\hbar }{i}\nabla +\frac{e}{c}\left[A_{B}\left(r\right)+{\hat{A}}_{\gamma }\left(r\right)\right]\right)}^{2}\right.\\ & \left.+V_{\mathrm{QD}}\left(r\right)\right]\hat{\psi }\left(r\right)+H_{Z}+{\hat{H}}_{ee}+\hbar \omega_{\gamma }{\hat{a}}^{†}\hat{a}. \end{array}$$

The electron field operator is $\hat{\psi }$, the magnetic vector potential is $A_{B}\left(r\right)=-By\hat{x}$ introduced in the Landau gauge, and ${\hat{A}}_{\gamma }$ is the vector potential of the photon cavity written as

(3) $${\hat{A}}_{\gamma }\left(r\right)=A\left(\hat{a}+{\hat{a}}^{†}\right)e.$$

Herein, *A* is the amplitude of the photon cavity field determined by the strength of electron–photon coupling $g_{\gamma }=eAa_{w}\Omega_{w}/c$, $e=e_{x}$ ($e_{y}$) when the photon field is parallel (perpendicular) to the direction of electron motion, and $\Omega_{w}$ refers to the effective confinement frequency determined by the external static magnetic field *B* and the bare confinement frequency $\Omega_{0}$, via $\Omega_{w}=\sqrt{\Omega_{0}^{2}+\omega_{c}^{2}}$, where $\omega_{c}=eB/m^{*}$. The photon creation and annihilation operators are represented by ${\hat{a}}^{†}$ and $\hat{a}$, respectively.

The second part of Equation (2) is the Zeeman Hamiltonian, which is $H_{Z}=\pm g^{*}\mu_{B}B/2$, with $\mu_{B}$ being the Bohr magneton and $g^{*}=-0.44$ the effective g-factor for GaAs. The Zeeman term shows the interplay between the electron magnetic moment and the magnetic field. The weak magnetic field here is to lift the spin degeneracy, which otherwise may create numerical difficulties. The external magnetic field and the parabolic confinement in the *y*-direction define a characteristic length scale, the effective confinement or magnetic length $a_{w}={\left(\hbar /\left(m^{*}\Omega_{w}\right)\right)}^{1/2}$. In addition, the third part of Equation (2) (${\hat{H}}_{ee}$) indicates the Coulomb interaction in the QD system [32], and the last part is the quantized photon field, with $\hbar \omega_{\gamma }$ as the photon energy.

To investigate the transport characteristics of the total system in the steady-state, we use a projection formalism built on the density operator [20,21]. Before coupling the QD system to the leads, the density operator of the total system is given by the tensor product of the individual density operators $\hat{\rho }\left(t<t_{0}\right)={\hat{\rho }}_{L}{\hat{\rho }}_{R}{\hat{\rho }}_{S}\left(t<t_{0}\right)$, where ${\hat{\rho }}_{L}$ and ${\hat{\rho }}_{R}$ indicate the density operator of the left (L) and the right (R) leads, respectively. After coupling the QD system to the leads, one can find the reduced density operator ${\hat{\rho }}_{S}$ that introduces the state of the electrons in the QD system under the effect of the leads as

(4) $${\hat{\rho }}_{S}={\mathrm{Tr}}_{L,R}\left[\hat{\rho }\left(t\right)\right],$$

where the trace is over the Fock space of the leads. We derive the equation of motion for the reduced density operator as a non-Markovian integrodifferential equation with a kernel evaluated up to the second order in the system–lead coupling [33]. As we are interested in the long-time evolution and the steady state of the system, we further transform the equation into a corresponding Markovian equation for the reduced density operator of the QD system [22]:

(5) $$\begin{array}{cc} \partial_{t}{\hat{\rho }}_{S}\left(t\right)= & -\frac{i}{\hbar }\left[{\hat{H}}_{S},{\hat{\rho }}_{S}\left(t\right)\right]-\left\{\Lambda^{L}\left[{\hat{\rho }}_{S};t\right]+\Lambda^{R}\left[{\hat{\rho }}_{S};t\right]\right\}\\ & -\frac{\bar{\kappa }}{2\hbar }\left({\bar{n}}_{R}+1\right)\left\{2\alpha {\hat{\rho }}_{S}\alpha^{†}-\alpha^{†}\alpha {\hat{\rho }}_{S}-{\hat{\rho }}_{S}\alpha^{†}\alpha \right\}\\ & -\frac{\bar{\kappa }}{2\hbar }\left({\bar{n}}_{R}\right)\left\{2\alpha^{†}{\hat{\rho }}_{S}\alpha -\alpha \alpha^{†}{\hat{\rho }}_{S}-{\hat{\rho }}_{S}\alpha \alpha^{†}\right\}. \end{array}$$

Herein, $\Lambda^{L}$ and $\Lambda^{R}$ represent the “dissipation” processes caused by both leads, $\bar{\kappa }=1.0\times 10^{-5}$ meV is the photon decay constant, and ${\bar{n}}_{R}$ indicates the mean photon number of the reservoir. The second and the third lines in Equation (5) display the photon dissipation of the cavity. $\alpha^{†}$ (α) stands for the original operator in the non-interacting photon number basis, $a^{†}$ (*a*), transformed to the interacting electron photon basis using the rotating wave approximation [34], where care has been taken in constructing a non-white noise spectrum appropriate for strong electron–photon coupling [35,36,37].

The QD system is coupled to the two leads that play the role of electron reservoirs obeying the Fermi–Dirac distribution

(6) $$F_{L,R}={\left[1+\exp\left(\left(E-\mu_{L,R}\right)/\left(k_{B}T_{L,R}\right)\right)\right]}^{-1},$$

with $\mu_{L}$ ($\mu_{R}$) being the chemical potential of the left (right) lead, and $T_{L}$ and $T_{R}$ are the temperatures of the left and right leads, respectively. The Fermi distribution of the leads is included in both dissipation terms $\Lambda^{L}$ and $\Lambda^{R}$ [22].

We consider the chemical potential of the leads to be equal ($\mu_{L}=\mu_{R}$) here and the temperature of the left lead to be higher than the temperature of the right lead. Therefore, the temperature gradient generates thermoelectric current through the QD system coupled to the leads. The thermoelectric current from the left lead into the QD system, $I_{L}$, and the thermoelectric current from it into the right lead, $I_{R}$, can be introduced as

(7) $$I_{L,R}={\mathrm{Tr}}_{S}\left(\Lambda^{L,R}\left[{\hat{\rho }}_{S};t\right]Q\right).$$

The charge operator of the QD system is $Q=-e\sum_{i}d_{i}^{†}d_{i}$, and ${\hat{d}}^{†}\left(\hat{d}\right)$ is the electron creation (annihilation) operator of the central system.

### Results

The total system, the QD system, and the leads are considered to be in a GaAs heterostructure where the relative dielectric constant is κ=12.4 and the effective mass is $m^{*}=0.067m_{e}$ [38,39]. The electron confinement energy in both the QD system and the leads is considered to be $\hbar \Omega_{0}=\hbar \Omega_{L,R}=2.0$ meV, and the cyclotron energy is $\hbar \omega_{c}=0.172$ meV at the weak magnetic field B=0.1 T applied to the total system, leading to $a_{w}=23.8$ nm.

**Figure 2:**
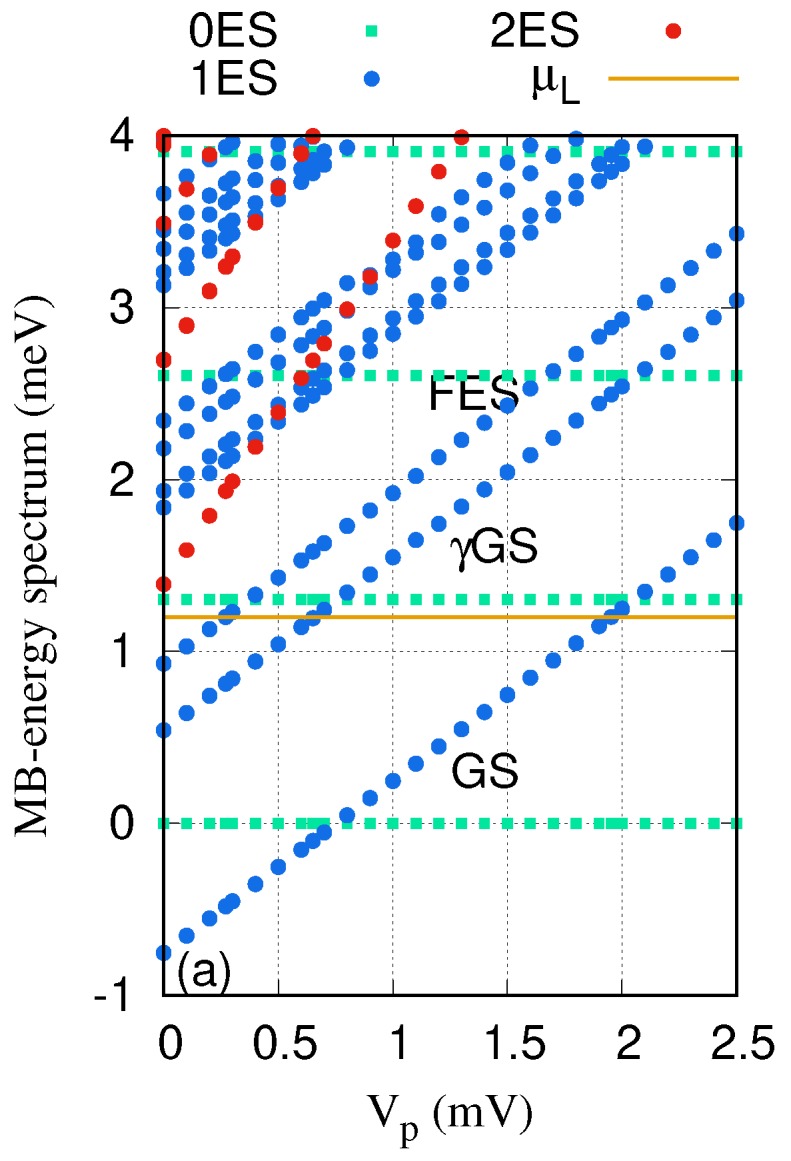
Many-body energy of the QD system versus the gate voltage ($V_{p}$), where 0ES (green squares) indicate zero-electron states, 1ES (blue circles) display one-electron states, and 2ES (red circles) refer to two-electron states. The golden line is the chemical potential of the leads where $\mu_{L}=\mu_{R}=\mu =1.2\;\mathrm{meV}$. GS indicates the one-electron ground-state, γGS is the one-photon replica of the one-electron ground-state, and FES is the one-electron first-excited state. The photon energy $\hbar \omega_{\gamma }=1.31$ meV, the electron–photon coupling strength is $g_{\gamma }=0.05$ meV, and and the photon field is linearly polarized in the *x*-direction. The magnetic field is B=0.1T, and $\hbar \Omega_{0}=2.0\;\mathrm{meV}$.

Figure 2 shows the Many-Body (MB) energy spectrum as a function of the plunger-gate voltage $V_{p}$ for the QD system coupled to the cavity. The golden horizontal line indicates the chemical potential of the leads $\mu_{L}=\mu_{R}=1.2$ meV. It is clearly seen that the ground-state (GS) at $V_{p}=1.95$ mV and the first-excited state (FES) at $V_{p}=0.271$ mV are touching (reaching) the chemical potential of the leads. Therefore, one can expect that these two states are responsible for the electron transport in the selected range of the gate voltage in the case of no photon cavity. The photon energy is assumed to be $\hbar \omega_{\gamma }=1.31$ meV, which is smaller than the energy spacing between the GS and the FES at $g_{\gamma }=0.05$ meV. Under these conditions, the QD system is not in resonance with the photon field. In addition to the two major states, the GS and the FES, there appear photon replica states. For instance, the lowest photon replica of the ground-state (γGS) appearing in the energy spectrum is in resonance with the chemical potential of the leads at $V_{p}=0.65$ meV. We note that the energy spectrum for the *x*- and the *y*-polarized photon fields is almost the same here.

**Figure 3:**
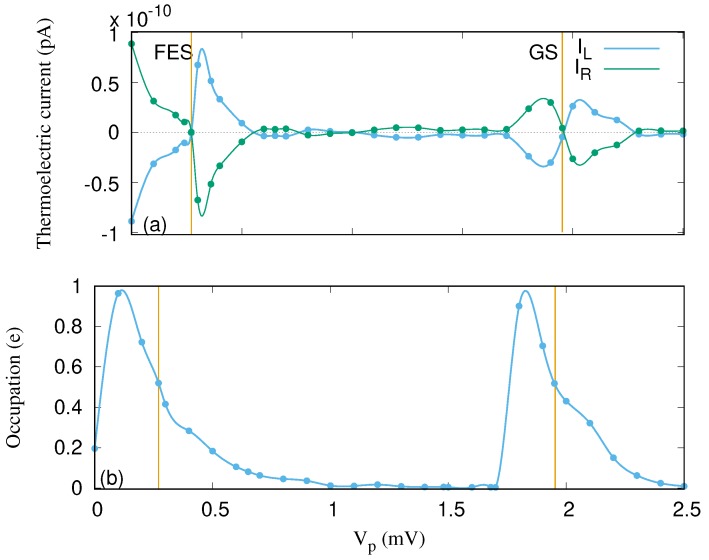
Thermoelectric current from the left lead to the QD system ($I_{L}$) and the thermoelectric current from the QD system to the right lead ($I_{R}$) (**a**) and occupation (**b**) versus the gate voltage $V_{p}$ for the QD system without the photon cavity. The temperature of the left (right) lead is fixed at $T_{L}=1.16$ K ($T_{R}=0.58$ K), implying thermal energy of 0.1 meV (0.05 meV). The chemical potential of the leads are fixed at $\mu_{L}=\mu_{R}=1.2$ meV. The golden vertical lines indicate the resonance condition for the ground-state (GS) at $V_{p}=1.95$ mV and the first-excited state (FES) at $V_{p}=0.271$ mV. The magnetic field is B=0.1T, and $\hbar \Omega_{0}=2.0\;\mathrm{meV}$.

To understand the properties of the thermoelectric current due to the temperature gradient, we start by considering the case of no photon cavity. In this case, the relevant states contributing to the transport are the original pure electron states, such as the GS and the FES. The left thermoelectric current $I_{L}$ into the QD system and the right thermoelectric current $I_{R}$ out of it for these two states as a function of the gate voltage are presented in Figure 3a. The left and the right thermoelectric currents are equal but with opposite signs, indicating the onset of a steady-state regime already at time just before $t=1\times 10^{8}$ ps, even though we follow the evolution to $t=1\times 10^{11}$ ps.

The thermoelectric current emerges due to the occupation or the difference between the two Fermi functions of the leads or the electron reservoirs. The thermoelectric current is observed when the Fermi functions of the leads have different widths but the same chemical potential. It can be described as follows. The thermoelectric current is zero in two situations: First, when the two Fermi functions of the leads or their occupations (see Figure 3b) are equal to 0.5 (half filling); second, when both Fermi functions or occupations are 0 or 1 (integer filling) [40,41]. As a result, the thermoelectric current is approximately zero at $V_{p}=0.271$ and 1.95 mV, corresponding to half filling of the FES and the GS, respectively [23]. The thermoelectric current is approximately zero at $V_{p}=1.8$ and 2.4 mV for an integer filling or occupation of 0 and 1 around the GS, respectively.

We note that the electron or charge occupation of the system is large when the GS or the FES are in or close to resonance with the the chemical potential of the leads. Without cavity photons, the charge almost exclusively resides in the corresponding resonant states and is vanishingly small for $V_{p}$ in the range between 1.0 and 1.7 mV. This can be understood keeping in mind that the temperatures $T_{L}$ and $T_{R}$ are very low, the GS localized in the quantum dot is very weakly coupled to the leads, and the electron density of states of the quasi-1D leads has a peak at the lowest sub-band bottom at 1.0 meV, while the GS is well below this value for this range of the $V_{p}$. The coupling to the leads depends on the spatial extension of the corresponding wave functions into the contact areas of width $a_{w}$ at the ends of each subsystem. In addition, the coupling depends on the electron affinity defined by $\exp(-|E_{a}-\epsilon \left(q\right)|/\Delta_{E})$, where $E_{a}$ stands for the states of the original single-electron basis for the central system, ϵ(q) is the energy spectrum of a lead, and $\Delta_{E}=0.5$ meV here [42]. The electron occupation or charge cumulation in the central system will be strongly affected by the cavity photon field, as will be reported below.

Let us now assume the situation where a photon field is applied to the QD system. In the off-resonant regime, the photon energy is considered to be $\hbar \omega_{\gamma }=1.31$ meV, which is smaller than the energy spacing between the two lowest states of the QD system ($E_{\mathrm{FES}}-E_{\mathrm{GS}}=1.682$ meV) for $g_{\gamma }=0.05$ meV and an *x*-polarized photon field.

**Figure 4:**
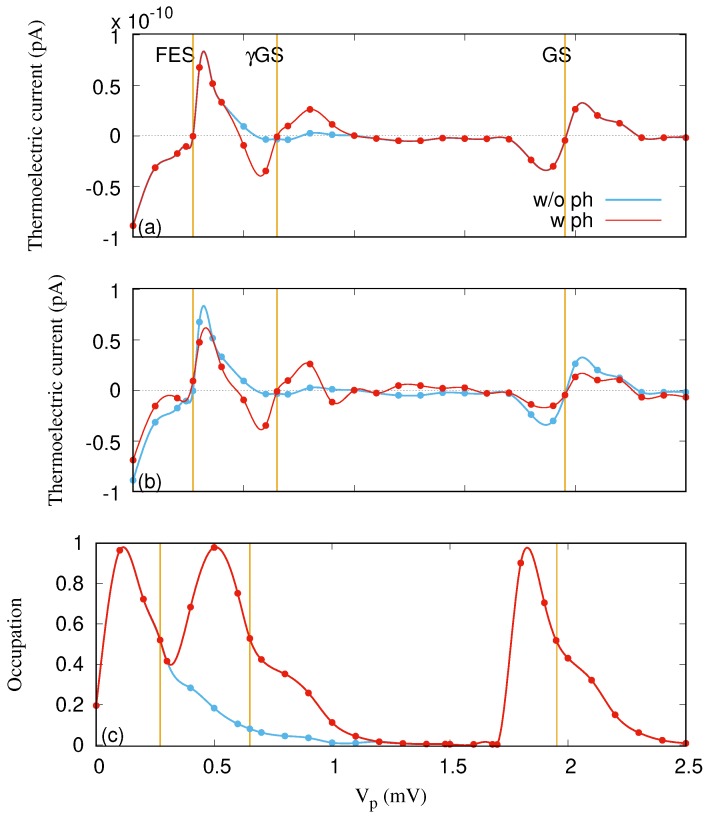
Thermoelectric current ($I_{L}$) in the case of ${\bar{n}}_{R}=0$ (**a**) and 1 (**b**), and electron occupation (**c**) for the QD system without (w/o ph) (blue color) and with (w ph) (red color) photon field. The photon energy is $\hbar \omega_{\gamma }=1.31$ meV (off-resonance regime), $g_{\gamma }=0.05$ meV, and the photon field is polarized in the *x*-direction. The temperature of the left lead is $T_{L}=1.16$ K and that of the right lead is $T_{R}=0.58$ K, which raises the thermal energy of the left lead to 0.1 meV and of the right lead to 0.05 meV. The chemical potential of the leads are fixed at $\mu_{L}=\mu_{R}=1.2$ meV. The golden vertical lines display the resonance condition for the GS at $V_{p}=1.95$ mV, the γGS at $V_{p}=0.65$ mV, and the FES at $V_{p}=0.271$ meV. The weak external magnetic field is B=0.1T, and $\hbar \Omega_{0}=2.0\;\mathrm{meV}$.

Figure 4 demonstrates the left thermoelectric current ($I_{L}$) for the off-resonant regime when the mean photon number is ${\bar{n}}_{R}=0$ (a) and 1 (b). In addition, its occupation versus the gate voltage is shown in Figure 4c. The occupation is almost the same for both cases of ${\bar{n}}_{R}=0$ and 1. Compared to the case of no photon field (blue color), extra current oscillation, from negative to positive, around the γGS at $V_{p}=0.65$ mV is observed in the presence of the photon field for both ${\bar{n}}_{R}=0$ and 1. The additional current oscillation arises due to a photon-assisted tunneling (PAT) [43]. An additional “peak” in the occupation around $V_{p}=0.65$ mV, shown in Figure 4c, is found corresponding to the extra current oscillation. The photon-assisted thermal transport has also been calculated for a simple two-level system using a Green function formalism [40,44]. We have not seen the extra thermal current peak in the transient regime [23,24]; however, the photon-assisted charge current peak can be clearly seen in the transient regime [32,45].

**Figure 5:**
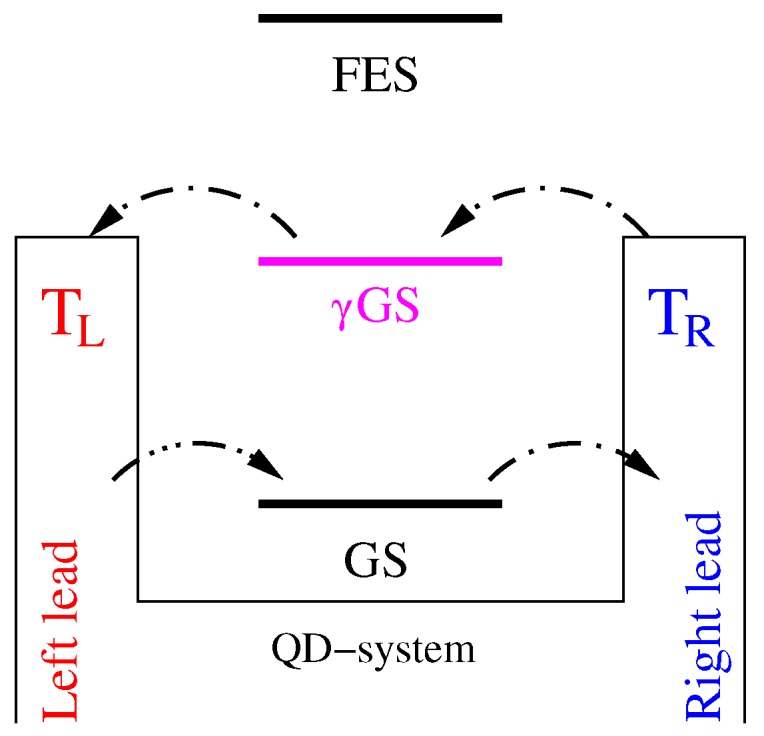
Diagram showing the photon-activated resonance energy levels and electron transition by the photon-induced processes via γGS.

We should mention that the thermoelectric current is almost unchanged when ${\bar{n}}_{R}=0$, and a suppression of thermoelectric current around the GS and FES for ${\bar{n}}_{R}=1$ is recorded due to the contribution of their photon replica states to the transport. The processes of current transport in the presence of the photon field is totally different here. For example, the contributed ratio of the GS to the transport is approximately 90% in the range $V_{p}=\left[1.8-2.2\right]$ mV where there is no photon field. But the GS is no longer the most active state that is responsible for the transport in the presence of the photon field. γGS together with the GS contribute to the transport in this range ($V_{p}=\left[1.8-2.2\right]$ mV), and the mechanism of thermal transport is totally different for these two states. Thermoelectric current flows from the left lead to the right lead through the GS. Surprisingly, the direction of current through the γGS is contrary, going from the right lead to the left lead, as is shown in Figure 5, irrespective of the direction of the thermal gradient. Therefore, the thermoelectric current is reduced. The reversed transport via the γGS can be related to the location of the chemical potential of the leads. For instance, if the chemical potential is located between the GS and the γGS, the GS (γGS) is located below (above) the Fermi function of the leads. In this case, the current must flow from the left lead to the right lead via the GS because it is below the Fermi function, and the opposite direction of flow may occur for the γGS as it is above the Fermi function.

**Figure 6:**
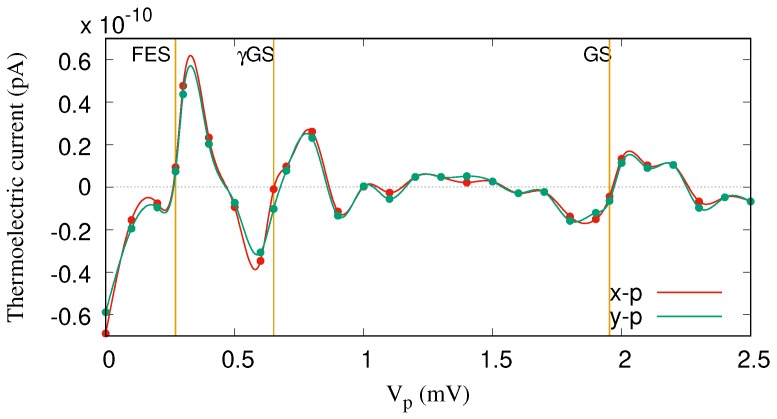
Thermoelectric current ($I_{L}$) for the QD system coupled to the photon field with *x*- (red color) and *y*-polarized (green color) photon fields. The photon energy is $\hbar \omega_{\gamma }=1.31$ meV (off-resonance regime), and $g_{\gamma }=0.05$ meV, ${\bar{n}}_{R}=1$. The temperature of the left and the right leads is fixed at $T_{L}=1.16$ K and $T_{R}=0.58$ K, respectively. The given temperatures imply that the thermal energy of the left lead is 0.1 meV and that of the right lead is 0.05 meV. The chemical potential of the leads are fixed at $\mu_{L}=\mu_{R}=1.2$ meV. The golden vertical lines indicate the resonance condition for the GS at $V_{p}=1.95$ mV, the γGS at $V_{p}=0.65$ mV, and the FES at $V_{p}=0.271$ meV. The weak external magnetic field is B=0.1T, and $\hbar \Omega_{0}=2.0\;\mathrm{meV}$.

The same explanation can be applied to the transport mechanism through the FES for the range $V_{p}=\left[0–0.5\right]$ mV, but instead of γGS, the one-photon replica of the first-excited state, γFES, contributes to the transport here. Figure 6 demonstrates the thermoelectric current versus the gate voltage for both *x*- (red color) and *y*-polarized photon fields (green color). It seems that the photon polarization for the off-resonance regime does not play an important role in the transport. The reason is that the location of the photon replica states in the MB energy spectrum is not sufficiently changed by tuning the photon polarization from the *x*- to the *y*-direction. Therefore, the contribution of the γGS to the transport is almost the same for both polarizations.

The total electron occupation in the central system, as displayed in Figure 4c, is similar to the results in Figure 3c except for the contribution around the γGS peak, but the partial occupation shows strong influences of the photon field. Around $V_{p}=1.8$ mV, the GS is occupied as before, but now around 1/4 of the charge resides in the FES. A total change takes place for $V_{p}$ in the 0.0–1.0 mV range. There, now 60–70% of the charge is in the GS and the rest is in the FES and the γGS. If the time evolution is analyzed, all of the charge enters the central system through the FES and the γGS, but the GS mainly gets occupied through slower radiative processes made possible by the photon field.

**Figure 7:**
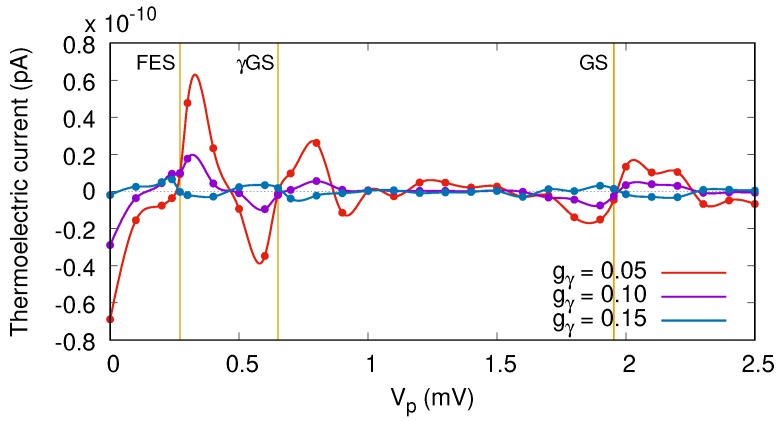
Thermoelectric current ($I_{L}$) versus gate voltage for the QD system coupled to the photon cavity with $g_{\gamma }=0.05$ (red color), 0.10 (magenta color), and 0.15 meV (navy blue color). The photon energy is $\hbar \omega_{\gamma }=1.31$ meV (off-resonance regime), ${\bar{n}}_{R}=1$, and the photon field is polarized in the direction of electron motion, the *x*-direction. The temperatures of the left and right leads are fixed at $T_{L}=1.16$ K and $T_{R}=0.58$ K, respectively. The chemical potential of the leads are fixed at $\mu_{L}=\mu_{R}=1.2$ meV. The golden vertical lines show the resonance condition for the GS at $V_{p}=1.95$ mV, the γGS at $V_{p}=0.65$ mV, and the FES at $V_{p}=0.271$ meV. The weak external magnetic field is B=0.1T, and $\hbar \Omega_{0}=2.0\;\mathrm{meV}$.

To recognize and further see the effects of the photon field on thermal transport, we display the thermoelectric current for a different electron–photon coupling strength $g_{\gamma }$ in Figure 7, where ${\bar{n}}_{R}=1$, and the photon field is polarized in the *x*-direction. By increasing the electron–photon coupling strength, the thermoelectric current is suppressed and a nearly zero current is recorded at $g_{\gamma }=0.15$ meV over the same interval of voltage as before. This happens because the contributing ratio of the GS and the γGS to transport are almost equal at a higher electron–photon coupling strength. As a result, the current is vanishing, and a plateau of nearly zero values is obtained.

**Figure 8:**
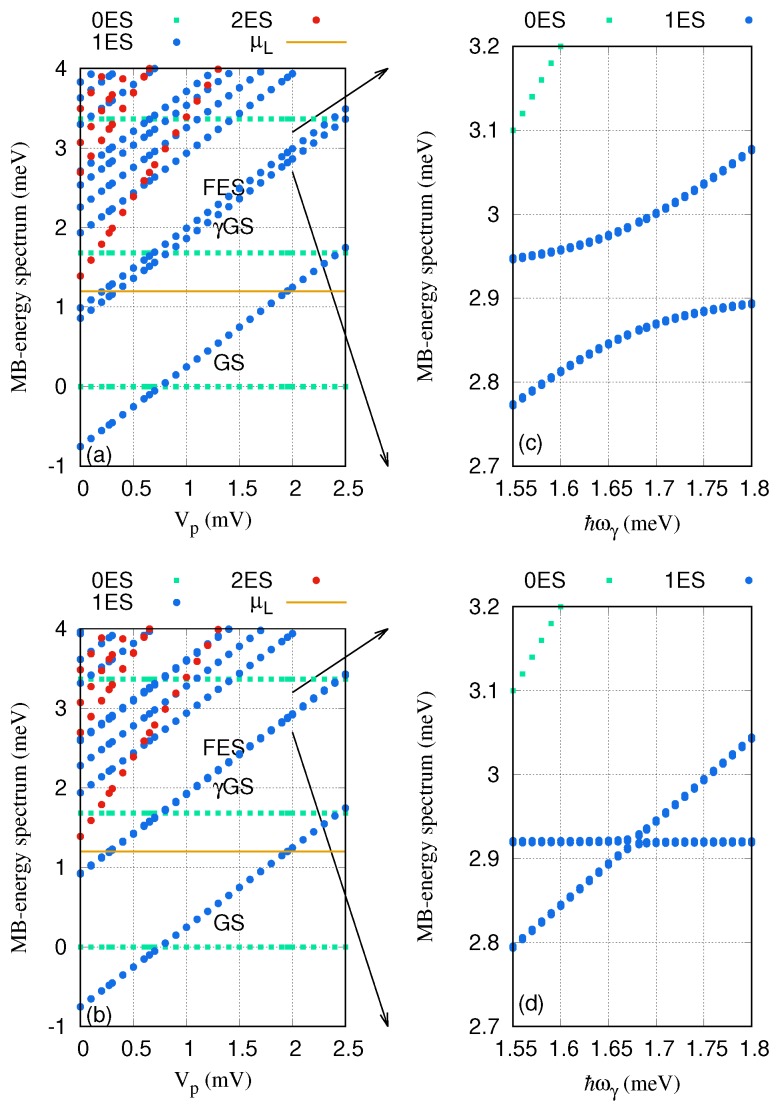
Many-body (MB) energy of the QD system versus the gate voltage ($V_{p}$), for the *x*- (**a**) and *y*-polarized (**b**) photon fields. The MB energy spectrum of the γGS and FES versus photon energy is plotted for *x*- (**c**) and *y*-polarized (**d**) photon fields. The 0ES (green squares) are zero-electron states, 1ES (blue circles) are one-electron states, and 2ES (red circles) are two-electron states. The golden line is the chemical potential of the leads where $\mu_{L}=\mu_{R}=\mu =1.2\;\mathrm{meV}$. GS indicates the one-electron ground-state, γGS is the one-photon replica of the one-electron ground-state, and FES is the one-electron first-excited state. The photon energy $\hbar \omega_{\gamma }=1.68$ meV, and $g_{\gamma }=0.05$ meV. The magnetic field is B=0.1T, and $\hbar \Omega_{0}=2.0\;\mathrm{meV}$.

We now investigate the resonant regime when the energy spacing between the GS and the FES of the QD system is approximately equal to the photon energy, $\hbar \omega_{\gamma }≃E_{\mathrm{FES}}-E_{\mathrm{GS}}$. The photon energy is considered to be $\hbar \omega_{\gamma }=1.68$ meV, the electron–photon coupling strength is $g_{\gamma }=0.05$ meV, and the mean photon number is ${\bar{n}}_{R}=1$. The MB energy spectrum is plotted against the gate voltage in Figure 8 for the *x*- (a) and *y*-polarization (b) of the photon field. The Rabi-splitting between the γGS and FES emerges and is larger for the *x*-polarized photon field. To confirm this, we display the MB energy spectrum of these two states as a function of the photon energy for *x*- (Figure 8c) and *y*-polarization (Figure 8d). The anti-crossings at the photon energy $\hbar \omega_{\gamma }=1.68$ meV indicates a Rabi-splitting, and it is quite small for the *y*-polarized photon field. The Rabi-splitting is larger for the x-polarization because the quantum dot system is an anisotropic system and the geometry of the QD system makes the charge densities of the states a bit more polarizable in that direction.

**Figure 9:**
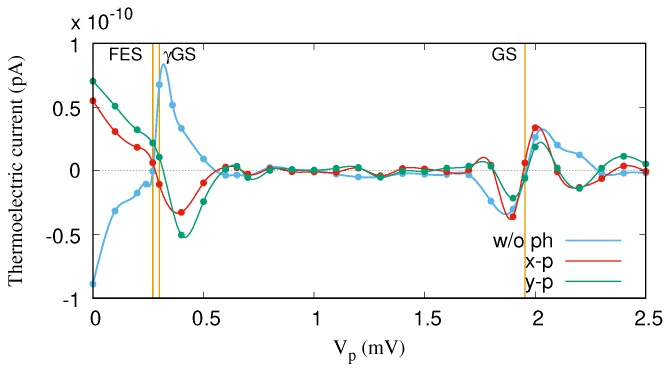
Thermoelectric current ($I_{L}$) for the QD system without (w/o ph) (blue color) and with (w ph) *x*- (red color) and *y*-polarized (green color) photon fields. The photon energy is $\hbar \omega_{\gamma }=1.68$ meV, $g_{\gamma }=0.05$ meV, and ${\bar{n}}_{R}=1$. The temperatures of the left and right leads are constant and fixed at $T_{L}=1.16$ K and $T_{R}=0.58$ K, respectively. The chemical potential of the leads are fixed at $\mu_{L}=\mu_{R}=1.2$ meV. The golden vertical lines indicate the resonance condition for the GS at $V_{p}=1.95$ mV, and the Rabi-splitting states between the γGS and the FES at $V_{p}=0.271$ meV. The weak external magnetic field is B=0.1T, and $\hbar \Omega_{0}=2.0\;\mathrm{meV}$.

The thermoelectric current for the on-resonant regime is shown in Figure 9. We find that the thermoelectric current through the GS is almost unchanged for both polarizations, but the characteristics of the thermoelectric current of the FES, which is in resonance with the γGS, is drastically modified. The effect of the resonant photon field is to invert the thermoelectric current from “positive” to “negative” values, or vice versa, around the FES at $V_{p}=0.271$ mV. The more γGS-like state at $V_{p}≃0.279$ mV participates in the transport of the electrons, with the more FES-like state leading to the current flip from “positive” to “negative” values. Furthermore, the first photon replica of the first-excited state (γFES) becomes active in the transport here. It should be noted that the current inversion is larger for the smaller Rabi-splitting in the *y*-polarized photon field. It indicates that the photon replica states have a major contribution in the transport; the resonance condition activates higher-lying states in the spectrum in the transport.

## 3. Conclusions

The characteristics of thermoelectric transport through a quantum dot embedded in a short quantum wire interacting with either off- or on-resonant cavity photon fields have been investigated in a steady-state regime. The QD system is considered to be connected to two electron reservoirs with different temperatures. The temperature gradient can accelerate electrons from either lead to the QD system, generating a thermoelectric current.

If a linearly polarized photon field is applied, the properties of the thermoelectric current are drastically changed. In the off-resonant regime, when the photon energy is smaller than the two lowest energy states of the QD system, an additional current peak is observed, which is only caused by photon-induced or photon-assisted transport. In addition, a plateau in the thermoelectric current is formed at a high electron–photon coupling strength. In the resonant regime, the effects of Rabi-splitting in the energy spectrum appears leading to an inversion of thermoelectric current. The inversion is more drastic for the smaller Rabi-splitting observed for a *y*-polarized photon field.

Even though we set out to investigate thermoelectric transport in the challenging regime of Coulomb blockades interrupted by narrow resonant peaks as the plunger gate voltage is changed, we observe that our approach indicates a mechanism that could be used in a thermoelectric inversion device.

## Abbreviations

The following abbreviations are used in this manuscript:

QD	Quantum dot
NEGF	Non-equilibrium Green’s function
$T_{L}$	Temperature of the left lead
$T_{R}$	Temperature of the right lead
MB	Many-body states
GS	Ground-state energy
γGS	One-photon replica of the ground-state
FES	First-excited state energy
